# Environmentally Friendly Gelatin/*β*-Cyclodextrin Composite Fiber Adsorbents for the Efficient Removal of Dyes from Wastewater

**DOI:** 10.3390/molecules23102473

**Published:** 2018-09-27

**Authors:** Yu Chen, Yanli Ma, Weipeng Lu, Yanchuan Guo, Yi Zhu, Haojun Lu, Yeping Song

**Affiliations:** 1 Key Laboratory of Photochemical Conversion and Optoelectronic Material, Technical Institute of Physics and Chemistry, Chinese Academy of Sciences, Beijing 100190, China; chenyubrc@mail.ipc.ac.cn; 2 University of Chinese Academy of Sciences, Beijing 100049, China; 3 Hangzhou Research Institute of Technical Institute of Physics and Chemistry, Chinese Academy of Sciences, Hangzhou 310018, China; mayanli@mail.ipc.ac.cn (Y.M.); zhuyi@mail.ipc.ac.cn (Y.Z.); luhaojun@mail.ipc.ac.cn (H.L.); songyeping@mail.ipc.ac.cn (Y.S.)

**Keywords:** environmentally friendly, gelatin, *β*-CD, adsorption, electrospinning

## Abstract

In this paper, environmentally friendly gelatin/*β*-cyclodextrin (*β*-CD) composite fiber adsorbents prepared by electrospinning were used for the removal of dyes from wastewater. Fourier transform infrared spectroscopy (FTIR), scanning electron microscopy (SEM) and a universal materials tester were employed to characterize the internal structures, surface morphologies and mechanical strength of the composite fiber adsorbents. Additionally, the fiber was evaluated as an adsorbent for the removal of methylene blue (MB) from aqueous solution. The effects of the raw material ratio, pH, temperature, concentration and adsorption time were studied. The results show that the gelatin/*β*-CD composite fiber adsorbents possess excellent mechanical strength and high adsorption efficiency for MB. The adsorption equilibrium and adsorption kinetics are well-described by the Langmuir isotherm model and the pseudo-second-order kinetic model, respectively. The theoretical maximum adsorption capacity is 47.4 mg·g^−1^. Additionally, after nine successive desorption-adsorption cycles, the removal rate is still over 70%. Moreover, the gelatin/*β*-CD composite fiber adsorbents exhibit excellent adsorption capability for basic fuchsin, gentian violet, brilliant blue R and malachite green dyes. Therefore, owing to the characteristics of degradability, low cost and high-efficiency, the gelatin/*β*-CD composite fiber can be used as an efficient adsorbent for the removal of dyes from wastewater.

## 1. Introduction

Nowadays, with the advancement and technological improvement of the dyeing industry, dyes are widely used in cosmetics, leather, paper, printing, plastic and textile finishing. However, the water polluted by dyes poses a great threat to ecology and human health, because most of these dyes are not degradable, can resist aerobic digestion and are stable to heat, light and oxidizing agents. Therefore, the removal of dyes from wastewater is an urgent environmental problem. At present, various methods, such as adsorption, coagulation, electrochemistry, photocatalytic degradation and ultrafiltration, have been utilized to remove dyes from wastewater [1,2,3,4,5,6]. Among these processes, adsorption is one of the most attractive technologies due to its high efficiency, simplicity, low cost and simple technology [7]. Although many adsorbents, such as activated carbon [8], clay [9], silica [10] and other porous materials [11,12], have already been used in the removal of dyes from wastewater, nanofibrous absorbent materials prepared by electrospinning have attracted a great deal of attention in recent years. This is because the electrospinning technique is a facile and low-cost method to prepare nanofibers with high surface area and high porosity, small inter fibrous pore sizes, and high gas permeability [13,14,15]. Therefore, it has become a hot spot in the research of adsorbing materials for organic pollutants.

Recently, researchers have focused on modifying nanofibers by various functional groups (–NH_2_, –COO^−^), cavity materials and porous silica materials to improve their dye adsorption ability [16,17,18,19,20]. Among them, *β*-cyclodextrin (*β*-CD) has gained considerable attention in the modification of composite nanofibers for the adsorption of pollutants. The most outstanding feature of *β*-CD is its ability to form host-guest complexes with a wide range of organic waste molecules, due to a special hydrophobic internal cavity and a hydrophilic external surface [21,22,23,24]. In recent years, researchers have conducted many studies on *β*-CD-functionalized dye adsorbent materials. Kadam et al. used a composite polyacrylonitrile (PAN)/*β*-CD nanofiber membrane to capture air pollutants, including aerosols and volatile organic compounds (VOCs), which showed an excellent air filtration performance [25]. Schafer et al. prepared poly (ether sulfone) (PES) nanofibers with incorporated *β*-CD for micropollutant (MP) removal from water [26]. Zhao et al. demonstrated a feasible method in the preparation of a water-insoluble sericin/*β*-CD/poly (vinyl alcohol) composite nanofiber adsorbent by using electrospinning to remove the cationic dye methylene blue from aqueous solution [27]. Intensive research demonstrates that the composite *β*-CD nanofibers play an important role in the adsorption of dyes. However, it is noteworthy that polymers, such as polymethyl methacrylate, polystyrene, polyester and polyether sulfone, using in composite *β*-CD nanofibers, are non-degradable and thus can easily cause secondary pollution and have a low utilization ratio, which greatly restricts their applications for adsorption [28,29]. Therefore, it is of great value and urgency to search for composite nanofiber adsorption materials with a high degree of environmental friendliness and an excellent absorption performance.

As a peptide-based polymeric material, gelatin obtained from the partial hydrolysis of collagen is a very rich denatured biopolymer material in the animal field [30]. It has excellent ease of availability, broad resources of raw materials and low cost [31,32]. Moreover, as a kind of natural polymer, gelatin also has excellent degradability [33,34,35] in vivo and in vitro; thus, it is expected to replace traditional non-degradable materials in the fields of drug delivery, tissue engineering and food packaging [36,37]. In addition, gelatin is an amphoteric polymer that contains a large number of amino, carboxyl and hydroxyl groups, which can occur in gelatinization, and has a certain surface activity, such as electrostatic interactions and hydrogen bonds [38]. Therefore, due to the special structure and properties of gelatin, we assume that it can be used as an excellent adsorbent for the adsorption of dyes.

**Scheme 1:**
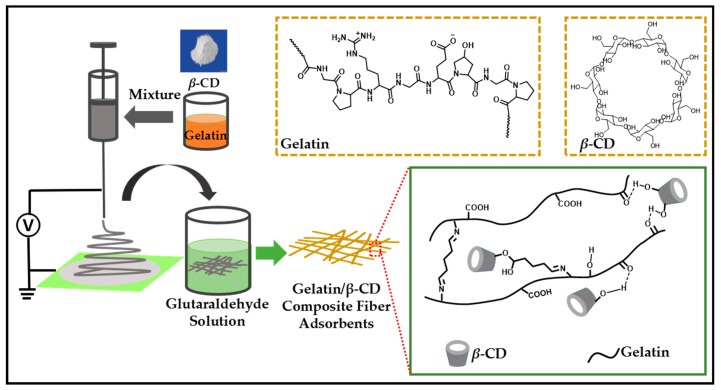
The preparation process and structure of gelatin/*β*-cyclodextrin (*β*-CD) composite fiber adsorbents.

In this study, environmentally friendly gelatin/*β*-CD composite fiber adsorbents were synthesized by electrospinning with trifluoroethanol as a solvent, and glutaraldehyde was used as a cross-linking agent for the cross-linking of composite fibers (Scheme 1). The adsorption properties of the gelatin/*β*-CD composite fiber adsorbents were studied using methylene blue (MB) dye as an example. Moreover, as far as we know, this is the first time that gelatin-based composite fibers have been used for dyes adsorption.

## 2. Results and Discussion

### 2.1. Morphologies of the Gelatin/β-CD Composite Fiber Adsorbents

**Figure 1:**
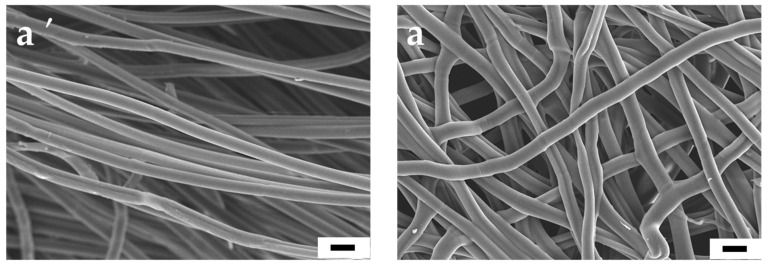

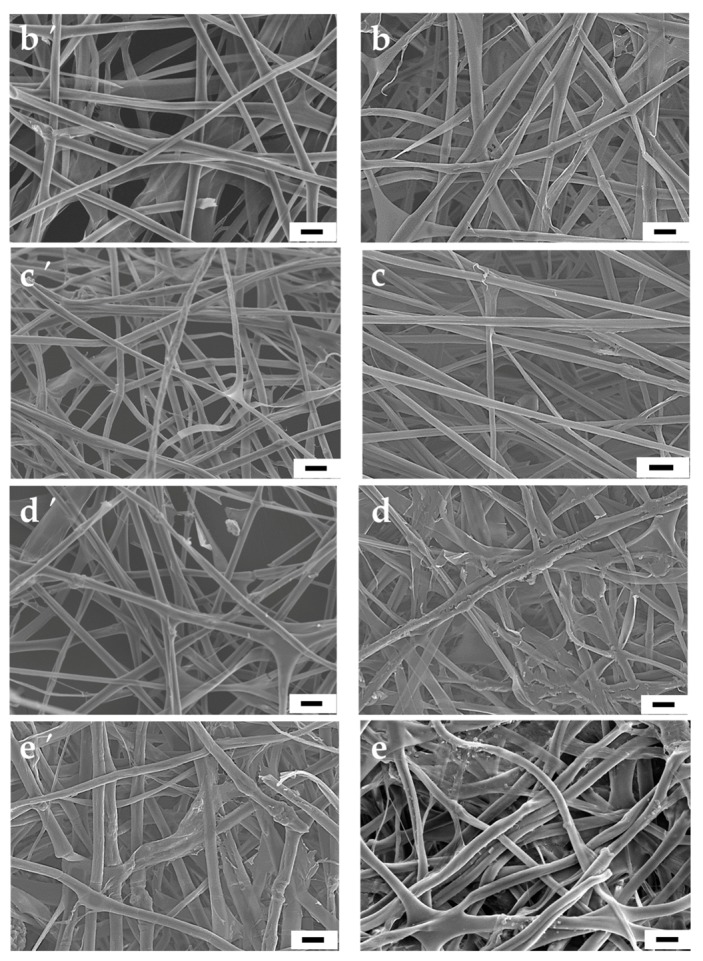
Field emission scanning electron microscope (FESEM) images of S0′ (**a**′), S1′ (**b**′), S2′ (**c**′), S3′ (**d**′) and S4′ (**e**′); S0 (**a**), S1 (**b**), S2 (**c**), S3 (**d**) and S4 (**e**) (the scale bar is 10 μM).

In order to investigate the morphologies and structures of fiber adsorbents, field emission scanning electron microscope (FESEM) measurements were performed. As shown in Figure 1, S0′, S1′, S2′, S3′ and S4′ represent the composite fiber adsorbents without cross-linking, which contain 0, 15, 30, 50 and 65% *β*-CD, respectively. S0, S1, S2, S3 and S4 represent S0′, S1′, S2′, S3′ and S4′ cross-linked by glutaraldehyde, respectively. The average diameters of S0′–S4′ were about 3.3, 3.5, 3.7, 3.9 and 4.2 μM, respectively. It can be seen that the pure gelatin fibers showed a uniform smooth surface, and the fiber thickness was consistent. However, because *β*-CD increased the viscosity of the electrospinning solution, the diameter of fibers appears to be thicker with the increase of the *β*-CD mass ratio in the composite fiber adsorbents. Furthermore, as the dyes’ adsorbent, gelatin/*β*-CD composite fibers need to have good physical/chemical stability in aqueous solution. Thus, glutaraldehyde was used as a cross-linking agent to improve the stability of the composite fiber adsorbents in a water atmosphere; the surface morphologies of S0–S4 are shown in Figure 1a–d. It demonstrates that the average diameter of the fiber increased after cross-linking, to about 3.7, 3.8, 4.0 and 4.3 μM, respectively. Moreover, the degrees of cross-linking and crimp between the fibers increased significantly and the cross-linking reaction caused the fibers to become denser and fuse at some intersections.

### 2.2. Fourier Transform Infrared Spectroscopy (FTIR) Characterization of Gelatin/β-CD Composite Fiber Adsorbents

**Figure 2:**
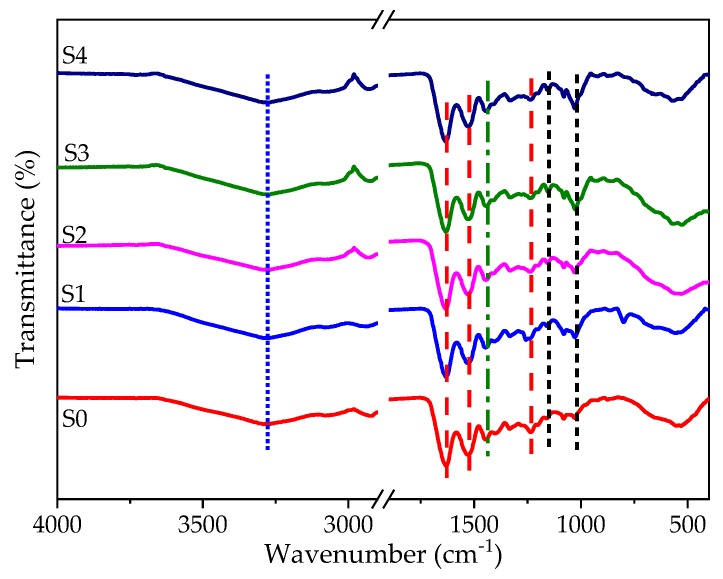
Fourier transform infrared spectroscopy (FTIR) spectrums of S0, S1, S2, S3 and S4.

To ascertain the chemical structures of gelatin/*β*-CD composite fiber adsorbents, FTIR was carried out, the results of which are shown in Figure 2. Due to the stretching of –OH and –NH, the gelatin molecule has a wide adsorption band at 3280 cm^−1^ [39]. Furthermore, the characteristic peaks at 1629, 1531 and 1237 cm^−1^ correspond to amide I, amide II and amide III, respectively, in which the amide I band is mainly attributed to the tensile vibration of –C=O, and the amide II and Ⅲ bands were caused by the bending vibration of -NH and the stretching vibration of –C–N, respectively [40]. In addition, due to the reaction between the aldehyde group of glutaraldehyde and the amino lysine residue of gelatin, the stretching vibration peak of the imide group (–CH=N) appears at 1450 cm^−1^ [41]. Simultaneously, attributed to the hydroxaldehyde condensation of *β*-CD with glutaraldehyde, new peaks appear in the range of 1150–1050 cm^−1^, caused by the stretching vibration (alkyl-substituted ether) of –C–O–C– and –C–O– [42]. Therefore, the above results confirm the successful cross-linking of gelatin with *β*-CD.

### 2.3. Mechanical Properties

**Figure 3:**
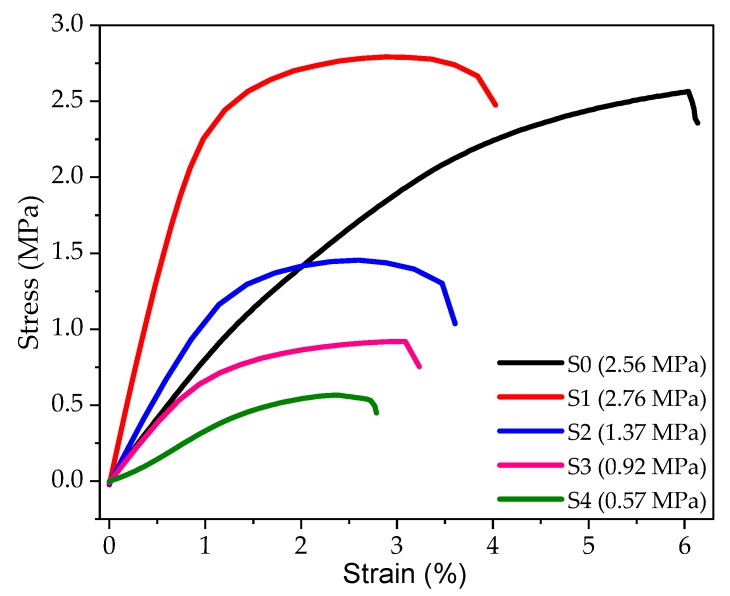
Tensile stress-strain curves of gelatin/*β*-CD and gelatin fiber adsorbents.

The mechanical properties of gelatin and gelatin/*β*-CD composite fiber adsorbents were evaluated using a universal materials tester; the stress-strain curves are shown in Figure 3. Compared to the pure gelatin fiber adsorbents (S0, 2.56 MPa), it is obvious that the tensile strength of the gelatin/*β*-CD composite fiber adsorbents (S1, 2.76 MPa) increased after the introduction of *β*-CD. The enhanced mechanical properties followed from the dense linkage of the cross-linking network between the carboxylic groups or hydroxyl groups of gelatin and the hydroxyl groups of *β*-CD, and hydrogen bonding between the gelatin matrix and the –OH group of *β*-CD. Furthermore, the cross-linking between gelatin and *β*-CD limited the movement of gelatin chains. However, as the content of *β*-CD increased, the tensile strength gradually decreased to 0.57 MPa. Although the uniform dispersion and good compatibility of gelatin and *β*-CD can improve the tensile strength of composite fiber adsorbents, excessive *β*-CD will accumulate and separate from the gelatin matrix, resulting in a gradual decline in tensile strength. Simultaneously, the elongation of adsorbents decreased gradually. After researching the literature [43,44,45,46,47], we think that although the addition of cyclodextrin leads to a decrease in the elongation at break and tensile strength, the adsorbent still shows good mechanical properties.

### 2.4. MB-Adsorption Behaviors

#### 2.4.1. Adsorption Kinetics of Gelatin/*β*-CD and Gelatin Fiber Adsorbents

**Figure 4:**
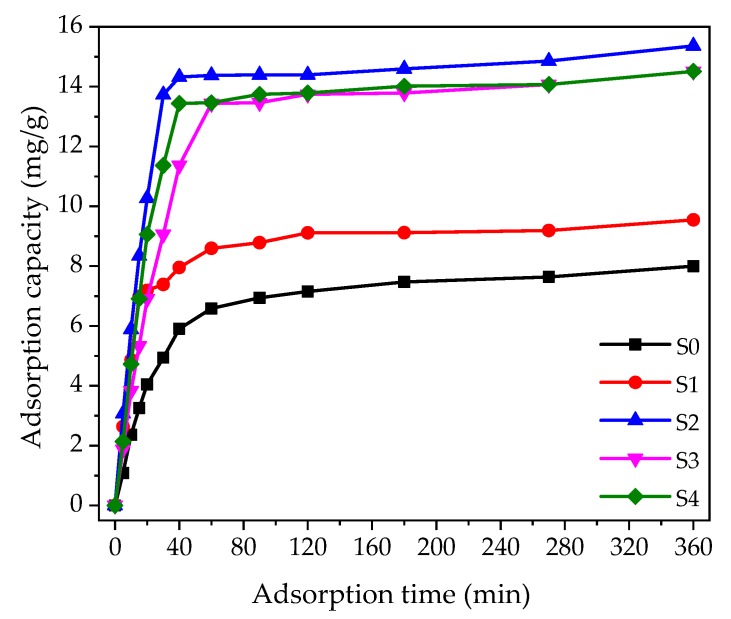
Kinetics of methylene blue (MB) adsorption onto gelatin and gelatin/*β*-CD composite fiber adsorbents.

To investigate the adsorption characteristics of gelatin/*β*-CD and gelatin fiber adsorbents for MB, adsorbent (10.0 mg) was immersed in 8.0 mL MB solution (20 mg·L^−1^, pH = 8.0), after which the absorbance-wavelength curve was measured at different adsorption times via a Ultraviolet-visible spectroscopy (UV-1800, SHIMADZU, Tokyo, Japan) implement. The concentration values at the maximum absorption wavelength were calculated by the Lambert-Beer law. Finally, the adsorption capacity values at different adsorption times were obtained by Equation (8). The adsorption capacity-adsorption time curves are shown in Figure 4. Attributed to many available adsorption sites, there was a rapid adsorption process in the first 40 min. With the extension of adsorption time, the adsorption sites decreased and MB molecules had to pass through the surface of the fibers into the fiber network, which resulted in a slower adsorption rate. The adsorption capacity became gradually saturated and reached the adsorption equilibrium [48]. In addition, as shown in Figure 4, since *β*-CD can form host-guest complexes with MB molecules, the adsorption property of gelatin/*β*-CD composite fiber adsorbents improved compared to the pure gelatin fiber adsorbent (S0). Due to the best adsorption performance of S2, all the subsequent experimental studies take S2 as the research object.

In order to evaluate the dynamic mechanism of the adsorption process of the gelatin/*β*-CD composite fiber adsorbents for MB, the pseudo-first-order and the pseudo-second-order kinetic models were used to fit the kinetic data, and the rate-determining step was judged by the fitting degree [49]. The pseudo-first-order and the pseudo-second-order dynamic equations are shown in Equations (1) and (2), respectively [50]:

(1) $$\log\left(q_{e}-q_{t}\right)=\log q_{e}-k_{1}t$$

(2) $$\frac{t}{q_{t}}=\frac{1}{k_{2}{q_{e}}^{2}}+\frac{t}{qe},$$

where $q_{t}$ and $q_{e}$ (mg·g^−1^) are the adsorption capacity at time *t* and equilibrium time, respectively, and *k*_1_ (min^−1^) and *k*_2_ (g·min^−1^·mg^−1^) are the pseudo-first-order and the pseudo-second order model rate constants, respectively.

**Figure 5:**
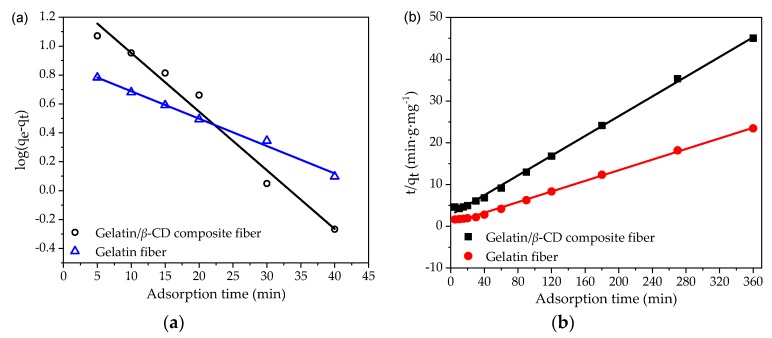
Adsorption kinetics of MB onto the gelatin and gelatin/*β*-CD composite fiber adsorbents based on the pseudo-first-order (**a**) and pseudo-second-order (**b**) kinetic model.

According to the pseudo-first-order and the pseudo-second-order kinetic equation, the curves of $\log\left(q_{e}-q_{t}\right)$ vs. *t* and *t*/*q_t_* vs. *t* were plotted and the fitting parameters of adsorption kinetic equations were obtained. Figure 5a,b shows the fitting diagrams of the pseudo-first-order and the pseudo-second-order dynamic model, respectively, and the obtained parameters are shown in Table 1. The fitting degree of the models was determined by the linear regression coefficient (R^2^). The relatively larger R^2^ value indicates that the model could successfully describe the adsorption kinetics of gelatin/*β*-CD composite fiber adsorbents for MB [51]. It can be seen from Figure 5 that the linear relationship of *t*/*q_t_* vs. *t* was better during the whole adsorption period, and that the R^2^ of the pseudo-second-order model was larger. Additionally, the calculated *q_e_* value was more consistent with the experimental data, indicating that the adsorption process followed the pseudo-second-order dynamic model. The adsorption of MB may be carried out by a surface exchange reaction until the surface functional sites are fully occupied, after which the MB molecules diffuse into the fiber network for further interactions, such as electrostatic forces, hydrogen bonds, van der Waals forces and inclusions effects.

#### 2.4.2. Adsorption Equilibrium

The distribution of dye molecules between the adsorbent and the equilibrium solution could be expressed using various equations. The Langmuir and Freundlich isotherms are commonly used; these formulas are shown in Equations (3) and (5) [52].

The equation of Langmuir is represented as follows:

(3) $$\frac{c_{e}}{q_{e}}=\frac{1}{K_{L}*q_{m}}+\frac{c_{e}}{q_{m}}$$

where *c_e_* (mg·L^−1^) is the equilibrium concentration of MB (mg·L^−1^) and *q_e_* (mg·g^−1^) is the equilibrium adsorption capacity of the MB adsorbed onto the gelatin/*β*-CD composite fiber adsorbents. $q_{m}$ is the maximum adsorption capacity of the adsorbent.

For the Langmuir adsorption process, the basic adsorption characteristics can be expressed in the separation factor (*R_L_*) which is defined by the following equation [53]:

(4) $$R_{L}=\frac{1}{1+c_{0}\times K_{L}}$$

where *c*_0_ is the initial concentration of MB (mg·L^−1^). The *R_L_* value indicates whether the adsorption process is irreversible (*R_L_* = 0), favorable (0 < *R_L_* < 1), linear (*R_L_* = 1) or unfavorable (*R_L_* > 1) [54].

The Freundlich isotherm is represented as follows:

(5) $$lnq_{e}=lnK_{F}+\frac{1}{nF}\times lnc_{e},$$

**Figure 6:**
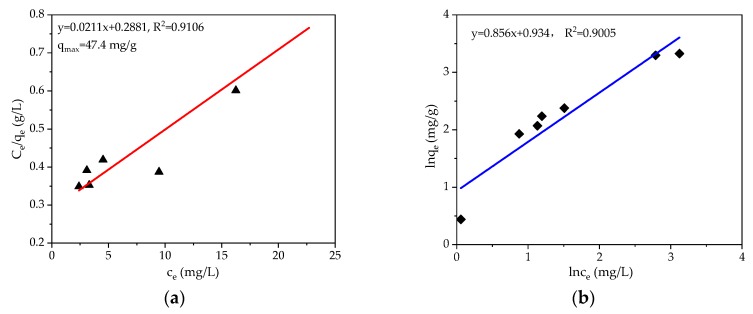
Adsorption isotherms of MB by gelatin/*β*-CD composite fiber adsorbents: Langmuir isotherm (**a**) and Freundlich isotherm (**b**).

According to the plot of *c_e_*/*q_e_* vs. *c_e_*, and $lnq_{e}$ vs. $lnc_{e}$, slop $1/q_{m}$ and intercept $\left(\frac{1}{K_{L}}\times q_{m}\right)$ were obtained where $q_{m}$ represents the theoretical monolayer maximum adsorption capacity. The results are shown in Figure 6a,b. It can be seen that the Langmuir isotherm is linear over the entire concentration range and has a good linear correlation coefficient (R^2^ = 0.9106), indicating that the data is consistent with the Langmuir relationship. The value of $q_{m}$ is 47.4 mg·g^−1^. Additionally, because the Langmuir equation assumes that the adsorbent surface is uniform, the Langmuir isotherm fitting results confirm the monolayer coverage of MB molecules and the uniform distribution of active sites onto the gelatin/*β*-CD composite fiber adsorbents.

**Figure 7:**
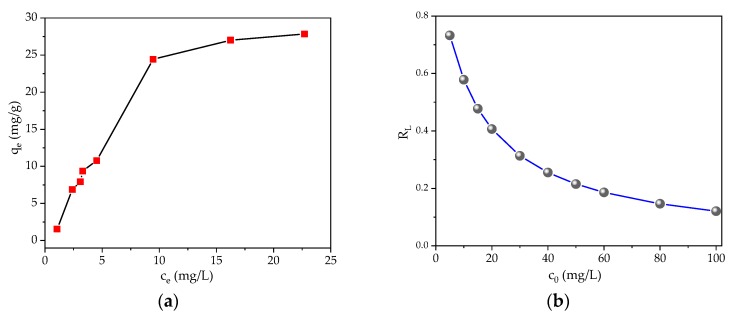
Adsorption isotherms of MB (**a**) and R_L_ for the adsorption of MB (**b**) onto gelatin/*β*-CD composite fiber adsorbents.

For the Langmuir adsorption process, the isotherm shape can be classified by the separation factor (*R_L_*) shown in Equation (4). The *R_L_* values of different initial dye concentrations are shown in Figure 7b. It can be observed that the value of *R_L_* is in the range of 0–1, confirming the adsorption process is “favorable”. Higher *R_L_* values at lower initial concentrations suggest that the adsorption is more favorable at lower MB concentrations.

#### 2.4.3. Effects of pH and Temperature

**Figure 8:**
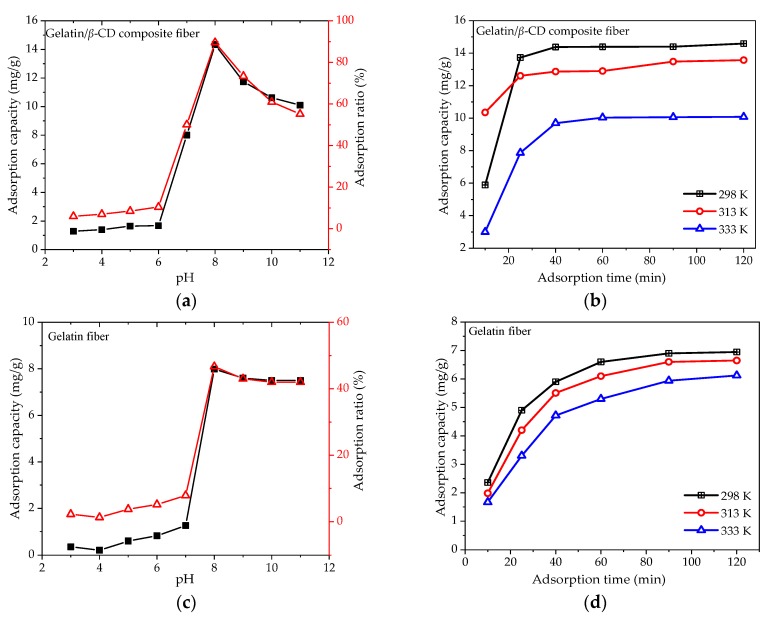
Effects of initial pH values (**a**,**c**) and temperature (**b**,**d**) on the adsorption property of MB.

The pH of the MB solution affects the surface charge of the adsorbent and the structure of dye molecules. Therefore, it is an essential factor for studying the adsorption performance. In this paper, the effects of the pH of the MB solution on the adsorption performance of gelatin/*β*-CD composite fiber adsorbents (S2) and gelatin fiber adsorbents (S0) were analyzed; the results are shown in Figure 8a,c. As can be seen from the trend of the curves, the adsorption capacity and adsorption efficiency increased with the increase of pH in the range of 3.0–8.0. This phenomenon can be explained by the electrostatic action. At low pH values, the electrostatic repulsion between protonated adsorption sites on composite fibers and protonated dimethylamine groups from MB restrict the interaction between MB molecules and composite fiber adsorbents. Therefore, the adsorption process is inhibited. At the same time, positive MB molecules have difficulty forming an inclusion effect via the *β*-CD cavity. With the increase of pH, positively charged MB molecules gradually change into a neutral state, and the carbonyl groups and hydroxyl groups with lone pairs on the gelatin/*β*-CD composite fiber adsorbents surface can interact with MB molecules through electrostatic force, hydrogen bonds and van der Waals force. Moreover, the adsorption capacity of an MB molecule increases significantly after binding with a *β*-CD cavity. When the pH value of MB solution exceeds 8.0, some of the hydroxyl and carboxyl groups on the composite fibers become deprotonated, and the density of electron clouds for groups containing nitrogen MB increases, resulting in the increase of the repellent force [51]. On the other hand, what cannot be ignored is the influence of the isoelectric point of the fibers. According to the literature [55,56], the isoelectric point (IEP) of the used gelatin (type B, basic-processed) is about 4.5–5.0. Therefore, when the pH of the MB solution is lower than the IEP, the gelatin surface presents as positively charged. Consequently, the positively charged gelatin rejects the protonated MB, which is not conducive to the adsorption of MB. When the pH of the MB solution increases, the gelatin surface changes into a neutral and negatively charged state and the electrostatic repulsion is reduced, resulting in an enhanced adsorption property. Compared to the adsorption capability of the gelatin fiber adsorbent (Figure 8c), the adsorption property of the gelatin/*β*-CD composite fiber adsorbent is enhanced due to the inclusion between the cavity of *β*-CD and MB. In conclusion, it can be inferred that the adsorption of gelatin/*β*-CD composite fiber adsorbents for MB is attributed to the forces of electrostatic force, hydrogen bonds, van der Waals force and inclusion effects.

Figure 8b,d show MB adsorption onto gelatin/*β*-CD composite fiber adsorbents and gelatin fiber adsorbents at different temperatures; the initial pH value is 8.0. The MB adsorption capacity (14.6–10.1 mg·g^−1^) presents a significant decreasing trend with the rise in temperature (298 k–333 k). The reason may be that the processes of MB adsorption onto gelatin/*β*-CD composite fiber adsorbents and gelatin fiber adsorbents are exothermic. Therefore, the increase of temperature is not beneficial to the positive movement of the adsorption process. The results suggest that MB adsorption on the adsorbent is favored at lower temperatures in the range of 20–60 °C.

#### 2.4.4. Recyclability of Gelatin/*β*-CD Composite Fiber Adsorbents

**Figure 9:**
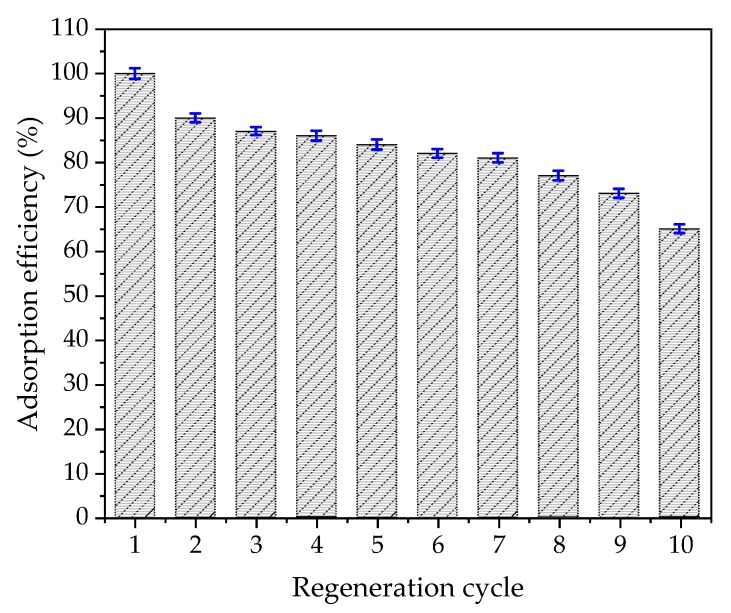
Desorption-adsorption cycles (adsorption efficiency of the first regeneration cycle was regarded as “100%”).

The removal of MB from electrospun gelatin/*β*-CD composite fiber adsorbents and the re-fixing of MB are the pivotal issues of adsorbent regeneration. Therefore, this paper evaluates the regeneration ability of gelatin/*β*-CD composite fiber adsorbents by measuring the adsorption capacity of the desorbed gelatin/*β*-CD composite fiber adsorbents. The desorption-adsorption cycle’s results are shown in Figure 9, and the first adsorption capacity of MB is regarded as “100%”. It can be seen that the adsorption efficiency of gelatin/*β*-CD composite fiber adsorbents still reach 73 ± 1.0% after nine desorption-adsorption cycles, meaning that the obtained electrospun gelatin/*β*-CD composite fiber adsorbents possess good reusability.

#### 2.4.5. Adsorption of Other Dyes

**Figure 10:**
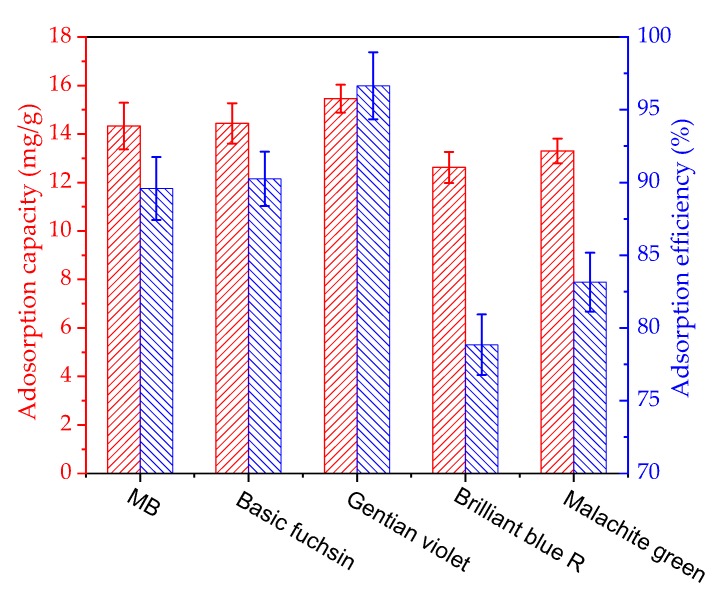
Adsorption capacity and efficiency of gelatin/*β*-CD fiber adsorbents for different dyes.

In order to inspect the adsorption performance of gelatin/*β*-CD composite fiber adsorbents with other dyes, the adsorption capacity and efficiency of basic fuchsin, gentian violet, brilliant blue R and malachite green were measured with the same adsorption conditions of MB; the results are shown in Figure 10. It can be seen that gelatin/*β*-CD composite fiber adsorbents possess high adsorption capacity (12.0–16.0 mg·g^−1^) and adsorption efficiency (>78%) for the four kinds of dyes. Based on the excellent adsorption properties for various dyes, gelatin/*β*-CD composite fiber adsorbents have great potential advantages as efficient adsorbents for the removal of dyes from wastewater.

### 2.5. The Adsorption and Desorption Mechanism

**Scheme 2:**
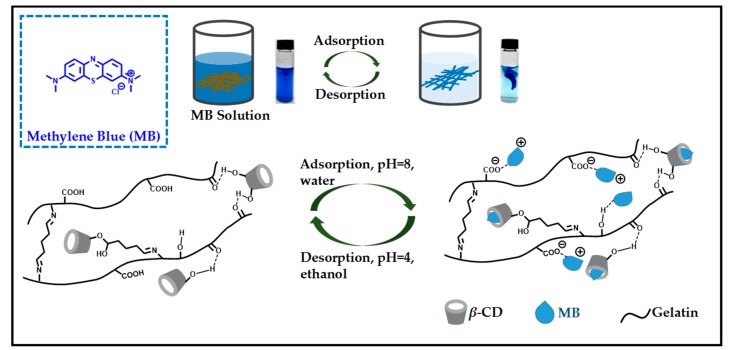
The adsorption and desorption mechanism of MB onto gelatin/*β*-CD composite fiber adsorbents.

**Figure 11:**
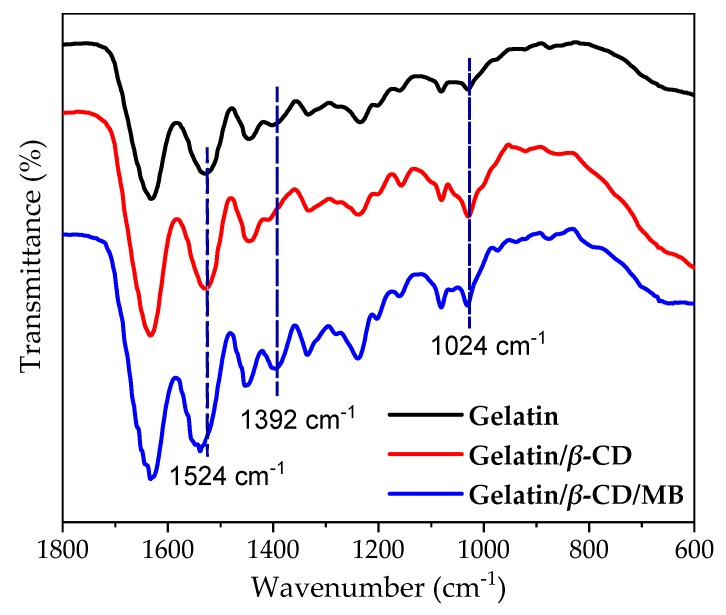
FTIR spectrum of gelatin fibers, gelatin/*β*-CD composite fiber adsorbents and gelatin/*β*-CD composite fiber adsorbents with MB adsorption.

Based on the above results, the adsorption and desorption mechanism is shown in Scheme 2. The adsorption of MB onto the gelatin/*β*-CD composite fiber adsorbents can be attributed to two processes: electrostatic interaction and complex host-guest interaction. Under electrostatic interaction, carboxyl groups in gelatin molecules can adsorb positively charged MB on the composite fiber adsorbents through positive and negative electrostatic interactions. For the host-guest interaction, we have mentioned that *β*-CD can be immobilized on gelatin molecules by cross-linking or hydrogen bonding; furthermore, *β*-CD can form host-guest complexes with MB molecules. Moreover, in order to further verify the successful adsorption of MB onto gelatin/*β*-CD fiber adsorbents, FTIR was used to characterize the changes of characteristic groups before and after the adsorption of MB. The results are shown in Figure 11. After adsorption, a new peak appeared at 1392 cm^−1^, which was attributed to the characteristic peak of –C=N in the MB molecule [32]. In addition, compared to the fibers without MB adsorption, a red shift occurred at the characteristic peaks at 1524 and 1024 cm^−1^, which might have been caused by the hydrogen bond between MB and gelatin and the host-guest interaction between *β*-CD and MB, respectively. For the desorption process, the acid environment of desorbed solution could destroy the inclusion effect between MB and the cavity of *β*-CD and the interactions between MB, gelatin and *β*-CD such as electrostatic force, hydrogen bonds and van der Waals force. Consequently, MB molecules are separated from fiber adsorbent carriers easily. The result is consistent with the weak absorption of MB under acidic conditions.

## 3. Materials and Methods

### 3.1. Materials

Gelatin (type B, basic-processed, obtained from bones, with a molecular weight of 100,000, viscosity value of 4.9 MPa·s^−1^) was provided by Dongbao Bio-Tech Co., Ltd. (Baotou, China). *β*-cyclodextrin (*β*-CD), 2,2,2-trifluoroethanol, glutaraldehyde (25 wt%), methylene blue trihydrate IND, basic fuchsin, gentian violet, brilliant blue R, malachite green and ethanol absolute were purchased from Titan Scientific Co., Ltd. (Shanghai, China). The deionized water used in the experiments possessed a resistivity of 18.2 MΩ·cm. All chemical reagents were analytical grade and employed without post-processing.

### 3.2. Preparation of Gelatin/β-CD Composite Fiber Adsorbents

For the preparation of gelatin/*β*-CD composite fiber adsorbents, gelatin (15%, *w*/*w*) was dissolved in 2,2,2-trifluoroethanol with constant stirring at 45 °C for 1 h, then *β*-CD (0%, 15%, 30%, 50% and 65% on the weight of gelatin, respectively) was added to the solution, and the homogeneous liquid was obtained by ultrasonic. For the electrospinning process, the solution was poured into the injection and connected to a high voltage power supply. The needle tip to the collector drum distance was fixed at 15 cm. The fibers were spun with a flow rate of 6 mL·h^−1^ at 17 kV. After electrospinning, the obtained fibers were soaked in glutaraldehyde ethanol solution (0.25%, *v*/*v*) for 12 h and then freeze-dried for 5 h for further use.

### 3.3. Morphologies of the Fiber Surface

The morphologies of fiber surface were observed by a field emission scanning electron microscope (FESEM, Vltra55, Carl Zeiss SMT Pte Ltd., Oberkochen, Germany) under an accelerating voltage of 3 kV. Samples were sprayed with gold coating. Images were registered at 20,000 and 60,000× magnifications.

### 3.4. FTIR Analysis

A spectrometer (Bruker Tensor II, Karlsruhe, Germany) with the digital attenuated total reflectance (ATR) accessory was used to obtain the FTIR absorbance spectra in the range of 4000–400 cm^−1^. Each spectrum was obtained at a resolution of 0.3 cm^−1^ with an average of 32 consecutive scans.

### 3.5. Mechanical Properties

Adsorbent thickness was the average value of four different measurement results on each specimen taken with a micrometer. Then, the texture analyzer (Instron, Norwood, CO, USA) was used to measure the tensile strength and percent elongation of adsorbents. The analysis was performed on 10 mm × 20 mm strips. The initial grip separation and crosshead speed were set at 12 mm and 50 mm·min^−1^, respectively. The percentage elongation (E%) and tensile strength of the adsorbents were calculated by the following equations:

(6) $$E\%=\frac{l-l_{0}}{l}\times 100\%$$

(7) $$\mathrm{Tensile}\;\mathrm{strength}=\frac{\mathrm{maximum}\;\mathrm{force}\;\left(N\right)}{\mathrm{thickness}\;\left(\mathrm{mm}\right)\times \mathrm{width}\;\left(\mathrm{mm}\right)}\times 100\%$$

where l_0_ and l represent the initial length and the length at the break point of the adsorbent, respectively. The result was an average of three measurements.

### 3.6. Adsorption of MB

Briefly, 10 mg composite fiber adsorbent was added into 8.0 mL MB solution (20.0 mg·L^−1^, pH = 8.0). The mixture was shaken at a constant speed of 120 rpm. After different time intervals, the adsorption mixture was centrifuged from its suspension; the concentration of MB was detected by a UV-vis spectrophotometer at the maximum adsorption wavelength of 664 nm. The dye concentration in the solution was calculated according to the standard curve equation, and the adsorption capacity *q_t_* (Equation (8)) and adsorption efficiency ρ (Equation (9)) were calculated by the change of methylene blue concentration before and after adsorption [57].

(8) $$q_{t}=\frac{(c_{0}-c_{t})\times V}{M}$$

(9) $$\rho =\frac{c_{0}-c_{t}}{c_{0}}\times 100\%,$$

where $c_{0}$ (mg·L^−1^) is the initial concentration of MB, $c_{t}$ (mg·L^−1^) is the MB concentration at time *t*, V is the volume of the testing solution (L) and M is the weight of the adsorbent (g).

### 3.7. Desorption and Recycling Study

In the desorption experiment, the composite fiber adsorbents adsorbed on MB were thoroughly cleaned with deionized water, and the adsorbents were then placed in ethanol solutions containing HCl (5% *v*/*v*). After desorption equilibrium, the adsorbents were washed several times with deionized water and freeze-dried. Finally, the adsorbents were reused in adsorption experiments (adsorbent, 10.0 mg; MB, 8.0 mL, 20.0 mg·L^−1^). The above processes were repeated 10 times to investigate the regeneration ability of the adsorbents.

## 4. Conclusions

A kind of environmentally friendly gelatin/*β*-CD composite fiber adsorbent was prepared by blending natural macromolecule gelatin with *β*-CD via the electrospinning technique. The composite fiber adsorbents exhibit excellent mechanical strength and high removal efficiency for MB. The adsorption isotherm and kinetics studies suggest that the equilibrium adsorption data and adsorption kinetics are well described by the Langmuir isotherm model and the pseudo-second-order kinetic model, respectively. The theoretical maximum adsorption capacity is 47.4 mg·g^−1^. It is worth noting that after nine cycles of desorption-adsorption regeneration, the composite fiber adsorbents still maintain high adsorption efficiency. Additionally, gelatin/*β*-CD composite fiber adsorbents possess excellent adsorption properties for basic fuchsin, gentian violet, brilliant blue R and malachite green dyes. The experimental results imply that the gelatin/*β*-CD composite fiber adsorbents have excellent adsorption application prospects for the removal of dyes from wastewater.

## Figures and Tables

**Table 1:** Kinetic parameters describing the adsorption of MB onto the gelatin and gelatin/*β*-CD composite fiber adsorbents based on the pseudo-first-order and pseudo-second-order kinetic models.

Adsorbent Composition	Pseudo-First-Order			Pseudo-Second-Order		
	$q_{e}/\mathrm{mg}\cdot g^{-1}$	$k_{1}/\min^{-1}$	$R^{2}$	$q_{e}/\mathrm{mg}\cdot g^{-1}$	$k_{1}/\min^{-1}$	$R^{2}$
Gelatin	7.5	0.0406	0.9716	8.5	0.0198	0.9977
Gelatin/*β*-CD	22.9	0.0189	0.9924	15.7	0.0015	0.9981
